# DNA gyrase could be a crucial regulatory factor for growth and survival of *Mycobacterium leprae*

**DOI:** 10.1038/s41598-019-47364-5

**Published:** 2019-07-25

**Authors:** Hyun Kim, Yasuo Fukutomi, Chie Nakajima, Youn Uck Kim, Shigetarou Mori, Keigo Shibayama, Noboru Nakata, Yasuhiko Suzuki

**Affiliations:** 1 Department of Bacteriology II, National Institute of Infectious Diseases, Musashi-Murayama, Tokyo, 208-0011 Japan; 2 Division of Bioresources, Hokkaido University Research Center for Zoonosis Control, Sapporo, 001-0020 Japan; 3 Antimicrobial Resistance Research Center, National Institute of Infectious Diseases, Higashimurayama, Tokyo 189-0002 Japan; 4 Department of Mycobacteriology, Leprosy Research Center, National Institute of Infectious Diseases, Higashimurayama, Tokyo 189-0002 Japan; 5 Global Station for Zoonosis Control, Global Institution for Collaborative Research and Education (GI-CoRE), Hokkaido University, Sapporo, Japan; 6 Department of Biomedical Sciences, Sun Moon University, A-San, 336-708 Republic of Korea

**Keywords:** Microbiology, Biochemistry

## Abstract

Leprosy, an important infectious disease in humans caused by *Mycobacterium leprae* (*Mle*), remains endemic in many countries. Notably, the pathogen cannot be cultured *in vitro*, except in mouse footpads *in vivo*. The molecular basis of these characteristics and the mechanisms remain unknown. Consequently, analysis of *Mle* growth and survival is urgently needed to develop novel therapies against leprosy, including rapid, simple, and specific methods to detect infection. Here, we demonstrated the functional role and contribution of *Mle-*DNA gyrase, which regulates DNA topology, DNA replication, and chromosome segregation to promote bacterial growth and survival, in *Mle* growth and survival *in vitro* and *in vivo*. The optimum temperature for *Mle*-DNA gyrase activity was 30 °C. When the DNA *gyrB-gyrA* genes in *Mycobacterium smegmatis* were replaced with the *Mle* gyrase genes by allelic exchange, the recombinants could not grow at 37 °C. Moreover, using radiorespirometry analysis for viability of *Mle* bacilli, we found that *Mle* growth was more vigorous at 25–30 °C than at 37 °C, but was inhibited above 40 °C. These results propose that DNA gyrase is a crucial factor for *Mle* growth and survival and its sensitivity to temperature may be exploited in heat-based treatment of leprosy.

## Introduction

Leprosy is a chronic human infectious disease caused by *Mycobacterium leprae* (*Mle*), with approximately 200,000 new cases reported worldwide every year^1^. Although the number of registered cases has dramatically decreased, the disease remains a public health issue, particularly in Asia, Latin America, and Africa^1,2^. A multidrug therapy (MDT) regimen, recommended as treatment by the World Health Organization in 1981^3,4^, has proved practical and successful against both multibacillary (MB; lepromatous pole) and paucibacillary (PB; tuberculoid pole) leprosy, resulting in a steep drop in the global incidence of leprosy^1,5^. MDT is based on treatment with dapsone (DDS)^6^, rifampin (RIF)^7^, and clofazimine^8^ as primary agents^3^. However, the emergence of multidrug-resistant leprosy has recently hampered treatment and global leprosy control^9–11^. Accordingly, novel therapies and drugs are needed.

*Mle* is an acid-fast, rod-shaped obligate intracellular bacillus^12^ that mainly colonises the coldest parts of the human body, including the skin, peripheral nerves, mucosa of the upper respiratory tract, eyes, and some other tissues. However, the precise mode of action in and mechanism of transmission to humans are unknown. *Mle* grows extremely slowly in comparison with other known bacteria, with a doubling time of 12–14 days^13,14^, and has not been cultivated on artificial medium *in vitro*. Indeed, *Mle* bacilli can only be cultured using Shepard’s mouse footpad system^15–17^, which requires 8–12 months and a relatively large number of seed mycobacteria. Consequently, the pathogen is difficult to investigate, culture, and/or detect to facilitate diagnosis or therapy.

The majority of eubacteria express two DNA topoisomerases II, namely, DNA gyrase and DNA topoisomerase IV; these enzymes are among a few clinically validated targets for antibacterial therapy^18^. Remarkably, *Mle* and *Mycobacterium tuberculosis* (*Mtb*) express only DNA gyrase^19–21^ from a *gyrB*-*gyrA* contig in the complete genome; this enzyme is the sole target of fluoroquinolones^19,22^. The catalytically active mycobacterial DNA gyrase, with a GyrA_2_GyrB_2_ heterotetrameric structure^23,24^, is an ATP-dependent enzyme that transiently cleaves and unwinds double-stranded DNA^24^ to catalyse DNA negative supercoiling. This enzyme is thus essential for efficient DNA replication, transcription, and recombination. Herein, we focused on the functional characteristics of *Mle*-DNA gyrase, with a view to understand the temperature sensitivity, growth, and survival of *Mle* bacilli.

In this study, we constructed and expressed the *Mle-*DNA gyrase as a recombinant enzyme, determined its activities *in vitro* and/or *in vivo* by radiorespirometry analysis for *Mle* viability and allelic exchange into other mycobacteria, and further evaluated its contribution to the growth and survival of *Mle* bacilli. Based on our results, we propose a novel therapeutic strategy—thermal therapy—against leprosy.

## Results

### Overexpression and purification of recombinant mycobacterial DNA gyrase

The construction of expression vectors encoding *Mle*- and *Mtb*-DNA gyrase is detailed in our previous reports^25–27^. Briefly, full-length DNA gyrase genes were inserted into the expression vectors pET-20a (+) and pET-19b to express His-tagged recombinant *gyrA* and *gyrB* downstream of the T7 promoter. The identity and integrity of these genes after cloning were confirmed by sequencing and alignment to reference sequences (data not shown); the results showed that mutations were not introduced during polymerase chain reaction (PCR). Milligram quantities of highly pure (>95%) recombinant proteins were obtained and were confirmed by sodium dodecyl sulfate polyacrylamide gel electrophoresis (SDS-PAGE) to be of the expected molecular size (Suppl. Fig. 1). Importantly, the preparations were free of contaminating *Escherichia coli* topoisomerase, as shown by the lack of supercoiling activity in assays using either GyrA or GyrB alone (data not shown).

### Determination of enzymatic activities using ATP-dependent DNA supercoiling assays

**Figure 1:**
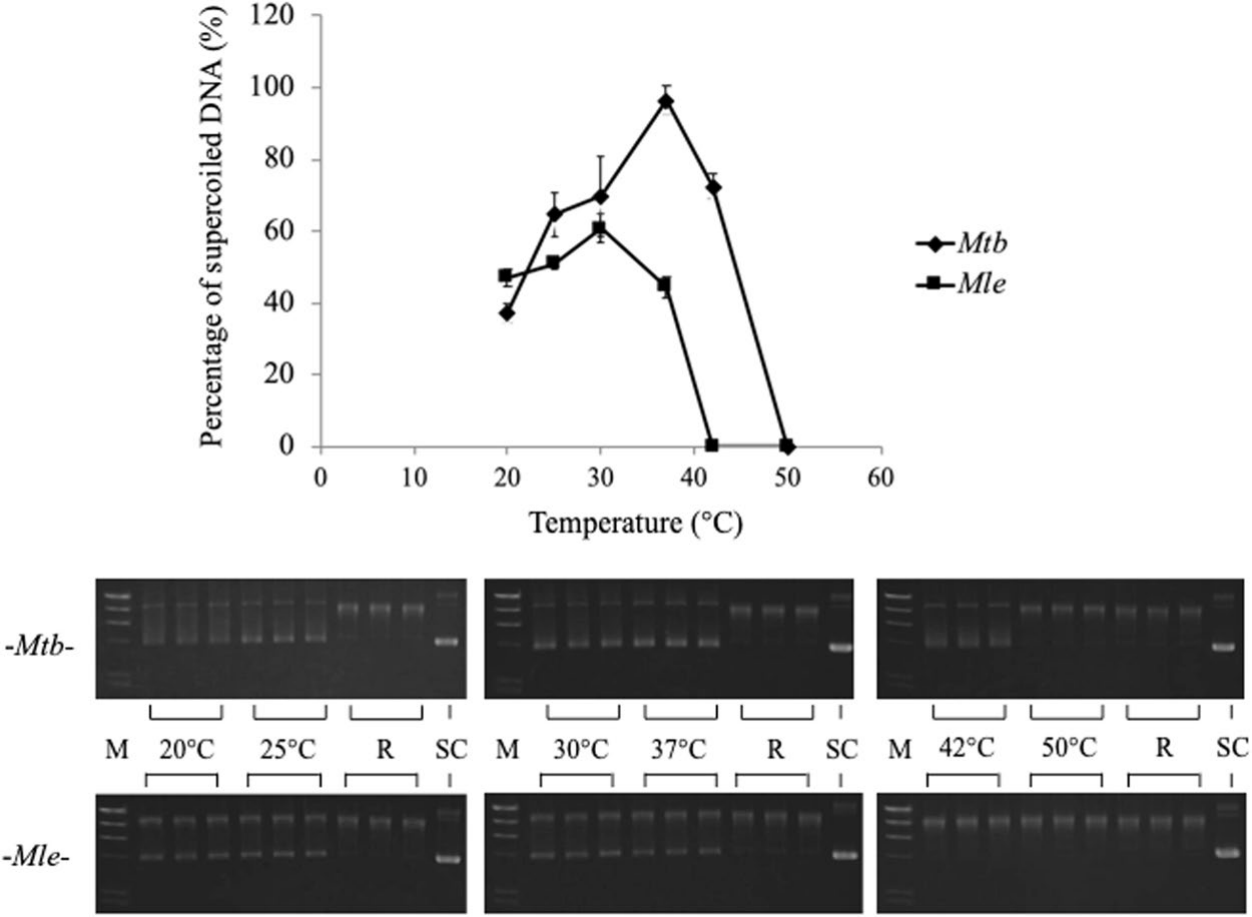
Temperature dependence of *Mle-* and *Mtb-*DNA gyrase activity. Relaxed pBR322 DNA (0.3 μg) was incubated at 20 °C, 25 °C, 30 °C, 37 °C, 42 °C, and 50 °C with DNA gyrases reconstituted from 3 μM of recombinant GyrA and GyrB. The results of electrophoresis are shown below the graph. Enzymes were assayed in triplicate. *Mle* and *Mtb* represent *M. leprae* and *M. tuberculosis*, respectively; M, R and SC represent *Hind*III DNA ladder, relaxed and supercoiled pBR322 DNA, used as a positive control for each enzyme activities.

**Figure 2:**
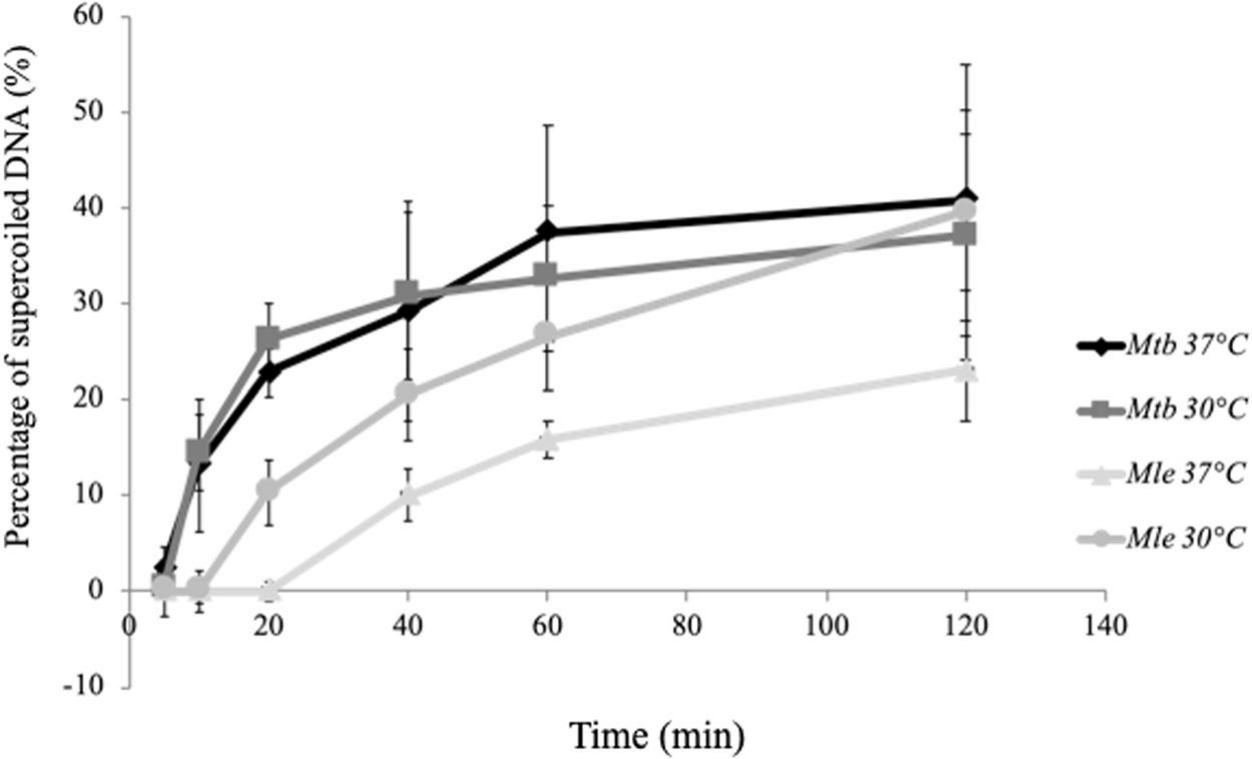
*Mle*- and *Mtb-*DNA gyrases were compared by examining ATP-dependent DNA supercoiling activity. Relaxed pBR322 DNA (0.3 μg) was incubated at 30 °C or 37 °C with DNA gyrase reconstituted from 3 μM of recombinant GyrA and GyrB. Mixture samples were examined for supercoiling activities at various time points (5, 10, 20, 40, 60, and 120 min) at 30 °C or 37 °C. The reactions were terminated, and the DNA products were analysed by electrophoresis on 1% agarose gels. Data are shown as the means and standard errors of at least three independent experiments; ◆ and ■, *Mtb*-DNA gyrase at 37 °C and 30 °C, respectively; ▲ and ●, *Mle*-DNA gyrase at 37 °C and 30 °C, respectively.

Combinations of DNA GyrA and GyrB were assayed for ATP-dependent DNA supercoiling activity using relaxed pBR322 DNA as the substrate (Figs 1 and 2, R and SC). We found that 3 μmol of GyrA (approximately 280 ng) and GyrB (approximately 240 ng) were sufficient for conversion of 100% of 0.3 μg of relaxed pBR322 DNA to its supercoiled DNA form in 60 min at varying temperatures (Fig. 1). The activities of *Mle-* and *Mtb-*DNA gyrases were highest at 30 °C and 37 °C (Fig. 2), respectively; all subsequent assays were conducted at these temperatures. The activities were lower at 37 °C and 42 °C, respectively, and undetectable at 42 °C and 50 °C, respectively (Fig. 1). The time-courses of reactions between 5 and 120 min at the corresponding optimum temperatures are plotted in Fig. 2. *Mle*-DNA gyrase was approximately 50% less active than *Mtb*-DNA gyrase, with a lag time of about 10–20 min in comparison (Fig. 2).

### Thermal inactivation of mycobacterial DNA gyrases

**Figure 3:**
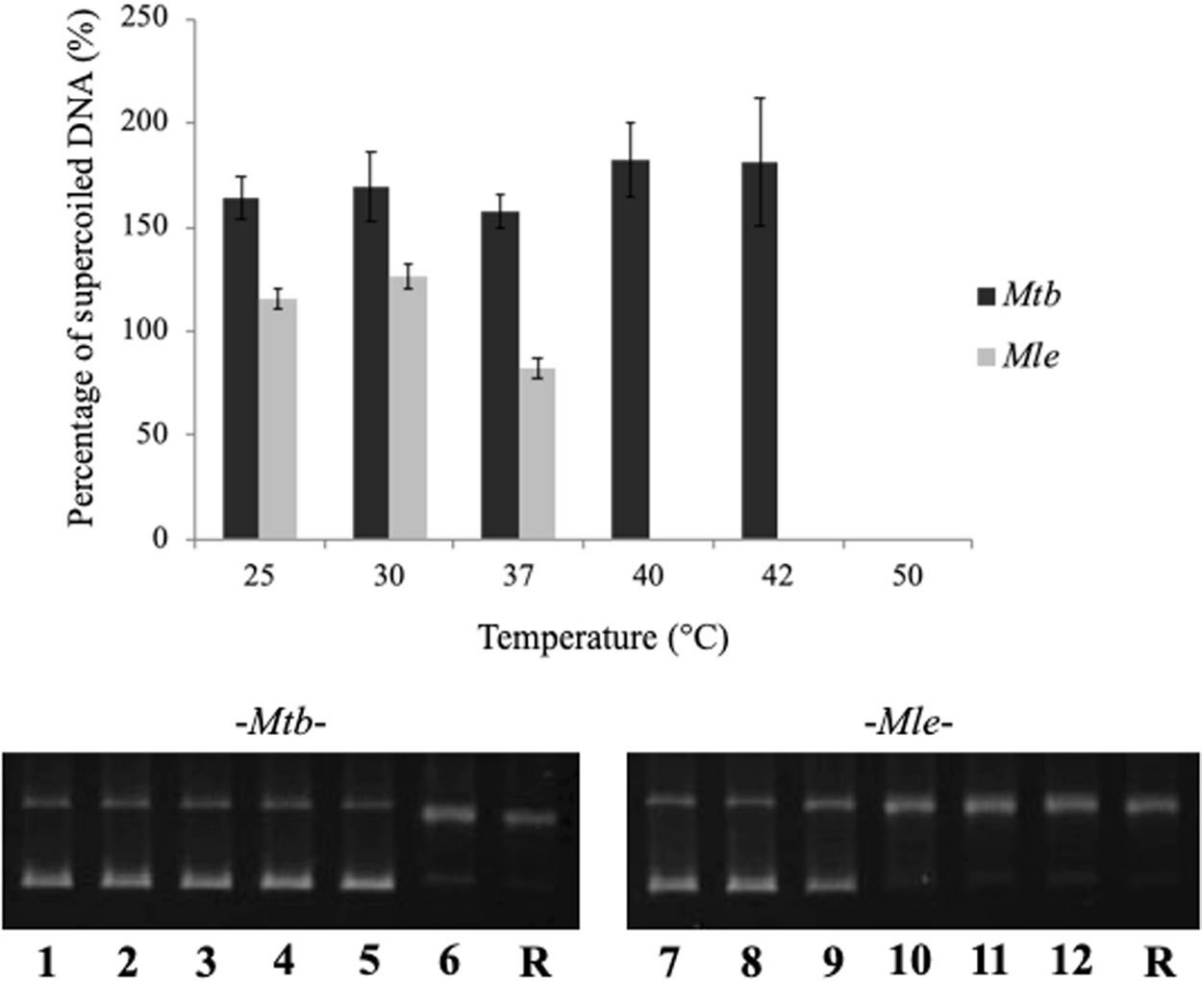
Thermal inactivation of *Mle*- and *Mtb*-DNA gyrases. DNA gyrases were inactivated for 1 h at various temperatures (25 °C, 30 °C, 37 °C, 40 °C, 42 °C, and 50 °C), cooled on ice for 1 h, and assayed for 1 h at 30 °C (*Mle*) or 37 °C (*Mtb*). Lanes 1 and 7, inactivation at 25 °C; lanes 2 and 8, inactivation at 30 °C; lanes 3 and 9, inactivation at 37 °C; lanes 4 and 10, inactivation at 40 °C; lanes 5 and 11, inactivation at 42 °C; lanes 6 and 12, inactivation at 50 °C. Black and gray bars indicate *Mtb-* and *Mle-*DNA gyrases. R denotes the relaxed pBR322 DNA, used as a positive control.

To assess thermal inactivation, DNA gyrases *in vitro* were incubated at various temperatures ranging from 25 °C to 50 °C (Fig. 3 and Suppl. Fig. 3), placed on ice, and assayed by supercoiling activity. Interestingly, residual activity was observed until at least 37 °C for *Mle*-DNA gyrase and until 42 °C for the *Mtb* enzyme (Fig. 3 and Suppl. Fig. 3). Furthermore, *Mle*-DNA gyrase at 37 °C was approximately 50% less active than for 30 °C. However, some inactivation was observed starting at 30 °C for the former, consistent with results shown in Fig. 1.

### Thermosensitivity analysis of *Mle* and *M. bovis* BCG strains by radiorespirometry

**Figure 4:**
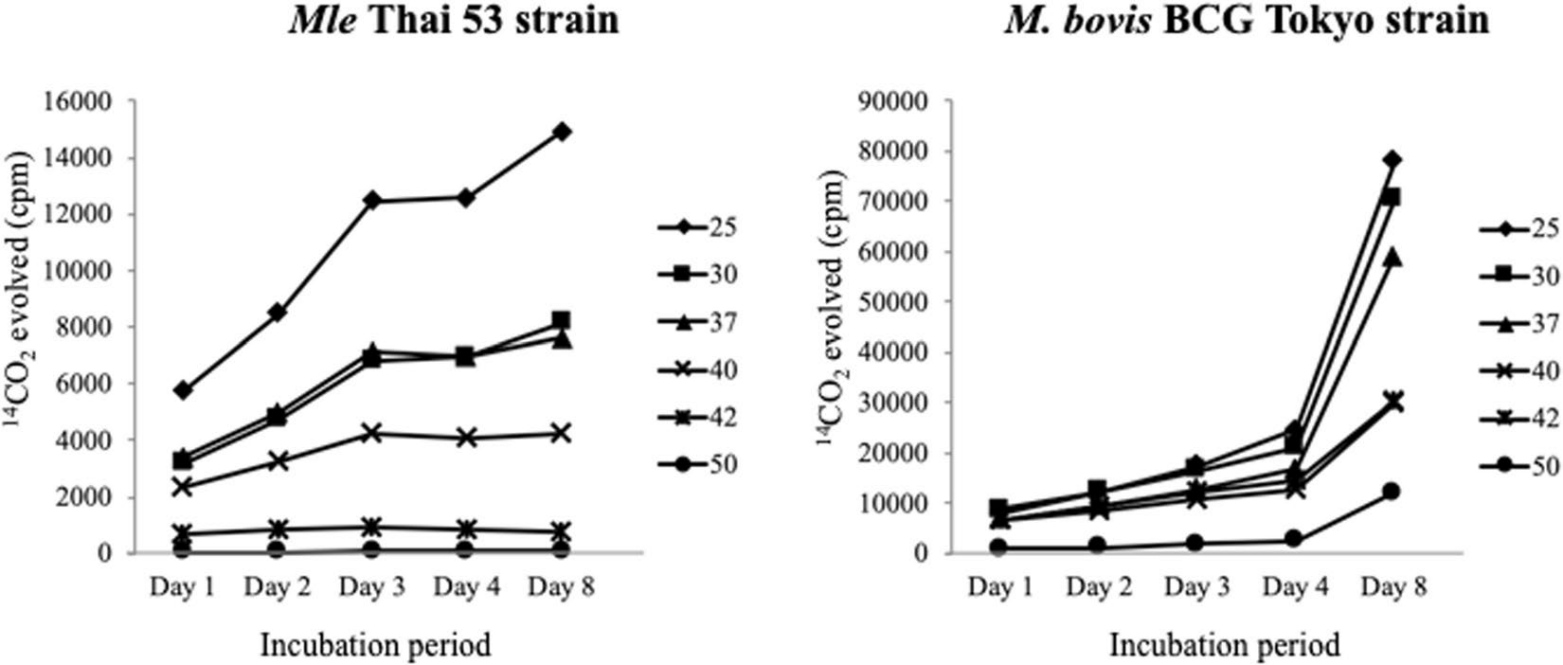
Thermosensitivity of *Mle-* and *M. bovis* BCG strains. *Mle* Thai-53 (left) and *M. bovis* BCG Tokyo (right) were pre-incubated for 1 h at 25 °C –50 °C, placed on ice for 1 h, and then grown in a BACTEC 12B medium at 30 °C and 37 °C, respectively. Viability was determined by radiorespirometry analysis, which evaluates the oxidation of ^14^C-palmitic acid to ^14^CO_2_. Total isotope release was analysed every day for 8 days; ◆, ■, ▲, ×, *, and ●, strains pre-incubated at 25 °C, 30 °C, 37 °C, 40 °C, 42 °C, and 50 °C, respectively.

Radiorespirometry analysis measures the cumulative release of ^14^CO_2_ from ^14^C-palmitate, and the CPM peak observed. Based on such an assay, viable *Mle* bacilli were observed to first undergo a lag phase, which was followed by a log phase with a CPM peak, ending in a plateau^28–31^. To assess temperature sensitivity, viable *Mle* and *M. bovis* BCG cells were first incubated at 20–50 °C, placed on ice for 1 h, and then assayed for viability at 30 °C and 37 °C. As shown in Fig. 4, *Mle* bacilli lost viability when incubated at temperatures above 40 °C. Inactivation was indistinguishable at 30 °C and 37 °C, although a few more cells appeared to survive at 30 °C. As expected, growth was higher in cells pre-incubated at 25 °C than in those pre-incubated at other temperatures (Fig. 4, left). In contrast, the growth of *M. bovis* BCG accelerated, beginning at day 4, regardless of prior exposure to any of the temperatures (Fig. 4, right). The observed viability of *Mle* was consistent with the temperature sensitivity of its DNA gyrase *in vitro* (Fig. 3).

### Influences of *Mle*-DNA gyrase on bacterial growth

**Figure 5:**
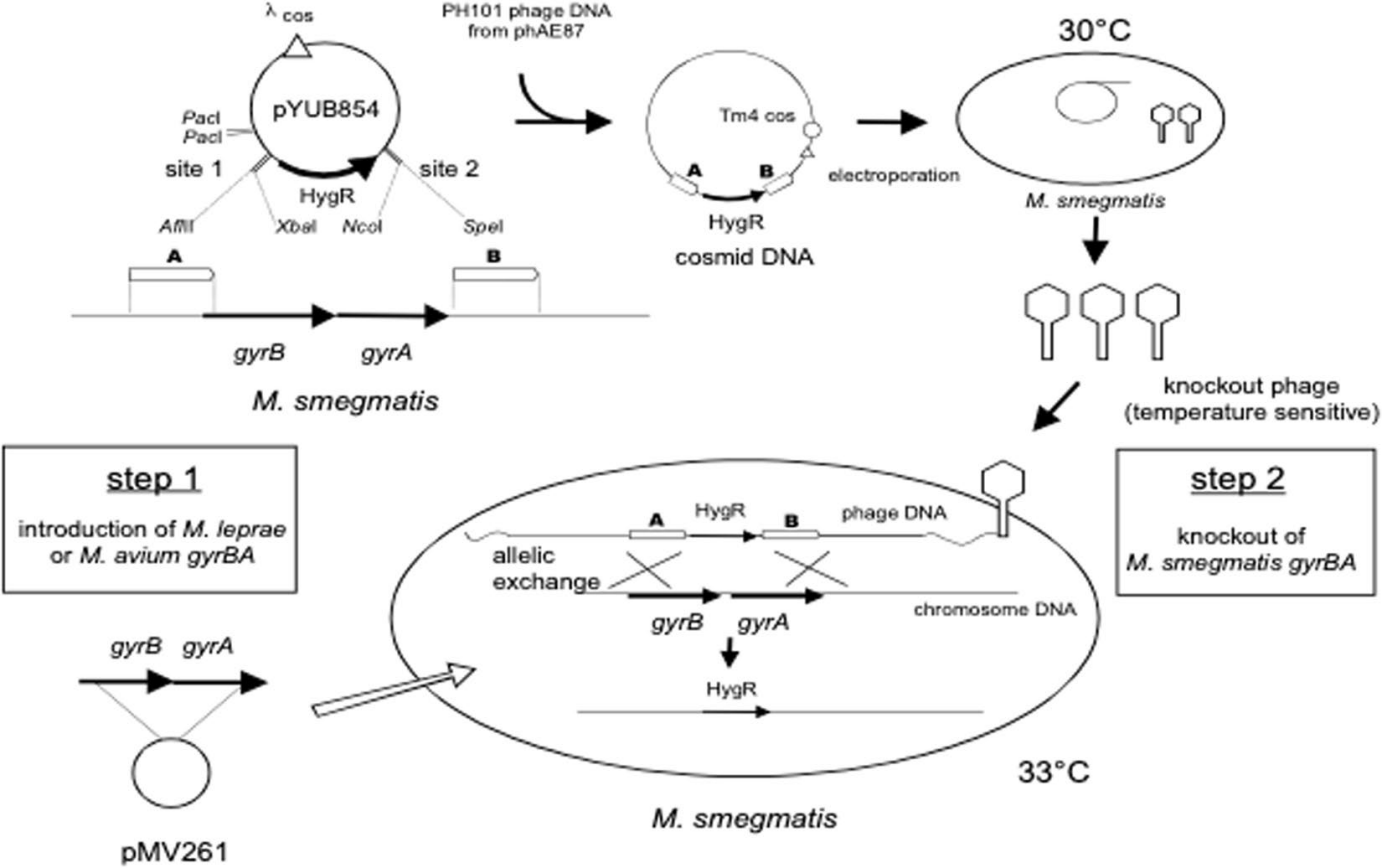
Scheme for construction of replacements *Mle*, *Mavi gyrB*-*gyrA* genes into *Msmeg* complete genome.

**Figure 6:**
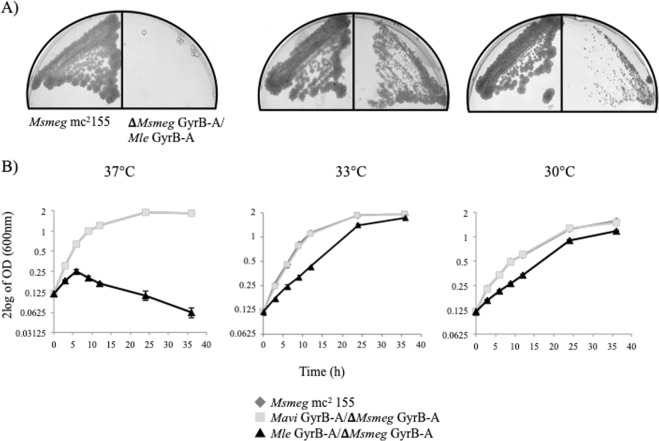
Growth of recombinant *Msmeg* strains relative to the parental strain mc^2^ 155. (**A**) Approximately 1 × 10^3^ bacterial cells were spread on 7H10 agar plates and incubated at 30 °C, 33 °C, and 37 °C. Left and right panels are *Msmeg* mc^2^ 155 and recombinant *Msmeg* with *Mle* GyrB-GyrA, respectively. (**B**) Growth rate of parental and recombinant mutants; ◆, ■, and ▲ indicate *Msm*eg mc^2^ 155, *Msmeg* with DNA *gyrB-gyrA* exchanged for *Mavi gyrB-gyrA* genes, and *Msmeg* with DNA *gyrB-gyrA* exchanged for *Mle gyrB-gyrA* genes.

To assess the impact of the temperature-sensitive activity of the *Mle-*DNA gyrase on other aspects of bacterial growth, we produced culturable mycobacterial strains having the *Mle gyrB-gyrA* genes instead of their own. *Mycobacterium smegmatis* (*Msmeg*) *gyrB-gyrA* genes were replaced with *Mle* or *Mycobacterium avium* (*Mavi*) *gyrB-gyrA* genes by allelic exchange (Fig. 5), as confirmed using PCR. At 37 °C, recombinants with *Mle*-DNA gyrase showed no colonies on 7H10 agar plates (Fig. 6A) and could not continue growing in 7H9 liquid medium (Fig. 6B). In contrast, cells thrived uniformly at 30 °C and 33 °C. Recombinants having *Mavi*-DNA gyrase had comparable growth rate and temperature sensitivity to the parental *Msmeg* mc^2^ 155 strain (colony data not shown), consistent with the similarity of its activity to that of the *Mtb* enzyme (Figs 1 and 3).

### Various combinations of *Mle*- and *Mtb*-DNA gyrase subunits

**Figure 7:**
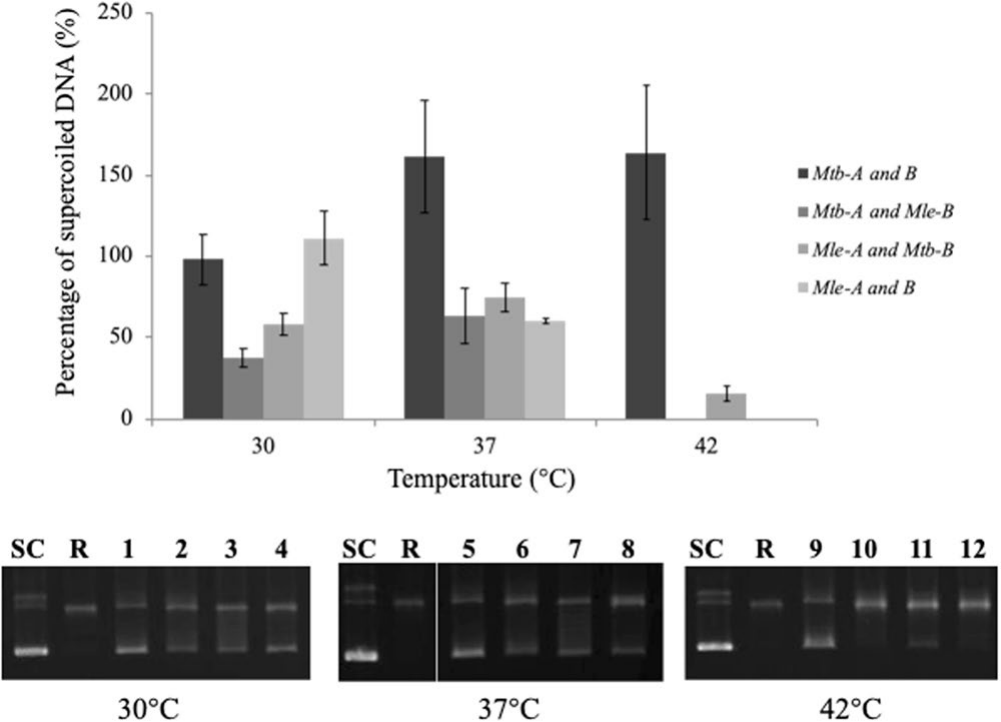
DNA supercoiling activity of various combinations of *Mle*- and *Mtb*-DNA gyrase subunits. Relaxed pBR322 DNA (0.3 μg) was incubated for 1 h at 30 °C, 37 °C, and 42 °C with DNA gyrase reconstituted from 3 μM of recombinant GyrA and GyrB subunits in various combinations. R and SC denote relaxed and supercoiled pBR322 DNA, used as a positive control for each enzyme activity.

To investigate the thermal sensitivity of each gyrase subunit, we combined the *Mle-* and *Mtb*-DNA gyrase subunits in various configurations, as *Mtb*-GyrA + *Mle-*GyrB or *Mtb*-GyrB + *Mle*-GyrA subunits (Fig. 7 and Suppl. Fig. 4). The mixed samples were incubated at different temperatures (data not shown 50 °C) and assayed or supercoiling activities. We observed that an enzyme consisting of *Mtb*-GyrA and *Mle-*GyrB showed no supercoiling activity at 42 °C, whereas a complex of *Mle*-GyrA and *Mtb-*GyrB was enzymatically active at the same temperature (Fig. 7 and Suppl. Fig. 4). Assays were performed in three replicates on the same day under identical conditions (Suppl. Fig. 4).

## Discussion

Slow growth, preference for low temperature, and resistance to *in vitro* culture are salient features of *Mle* bacillus. For example, the optimum temperature for this species was previously reported to be several degrees above and below 30 °C^29,31^, whereas other mycobacteria grow at 37–42 °C. Therefore, functional analysis of *Mle* is urgently needed to determine the factors critical for bacterial growth and survival. We speculated that DNA gyrase is one factor responsible for their growth, as this enzyme is essential for DNA replication, transcription, and recombination. Accordingly, we characterised the association of DNA gyrase with bacterial survival. To this end, the full-length DNA gyrases from *Mle* and *Mtb* were cloned, expressed, and purified from *E. coli*. We observed that the optimum reaction temperature for the recombinant *Mle* enzyme was 30 °C *in vitro*, which coincides with the optimum growth temperature for this species^29,31^.

To avoid handling *Mle*, which is not cultivable, we also replaced the DNA *gyrB-gyrA* genes in *Msmeg* with those of *Mle*, using a method we previously developed^32–34^. Because DNA gyrase is essential for bacterial growth and survival, we first introduced the *Mle*-DNA *gyrB-gyrA* genes into *Msmeg* using a multicopy shuttle plasmid vector (pMV261) before disrupting the endogenous *Msmeg-*DNA *gyrB-gyrA* genes (Fig. 5). The resulting mutant, which expresses *Mle*-DNA *gyrB-gyrA* genes instead of its own, grew at 30 °C and 33 °C, but not at 37 °C. These results suggested that the temperature dependence of *Mle* growth and survival could be explained by its thermosensitive DNA gyrase (Fig. 6).

We also found that *Mle*-DNA gyrase was inactivated at temperatures higher than 37 °C, whereas the *Mtb* enzyme was inactivated at temperatures above 42 °C. These results proposed that temperatures of greater than 37 °C may degrade or induce significant conformational changes in *Mle-*DNA gyrase, resulting in inactivity (Fig. 3).

To complement the *in vitro* experiment data, we analysed the growth rate of viable *Mle* bacilli by radiorespirometry analysis, which measures ^14^CO_2_ formation due to metabolism of ^14^C-palmitic acid^29,30,35^. We observed that temperatures above 40 °C were not suitable for *Mle* bacilli. Therefore, our findings suggested that DNA gyrase regulates *Mle* growth and survival (Fig. 4).

Indeed, mycobacterial DNA gyrase, which consists of two subunits (GyrA and GyrB) that combine to form the functional heterotetrameric A_2_B_2_ complex, is an essential type II topoisomerase. Although many studies on the crystal structures of these subunits have suggested possible functions^23,36–38^, to our knowledge, the temperature dependence of its catalytic activity has not been investigated. Therefore, we investigated the *Mle-* and *Mtb*-DNA gyrase subunits using various configurations (Fig. 7 and Suppl. Fig. 4), which results shown that the GyrB subunit appears to be temperature-sensitive, although the molecular basis of this sensitivity is unknown. To elucidate which GyrB domain is associated with temperature-sensitivity, the three-dimensional (3D) structure of the *Mle*-DNA GyrB subunit was predicted using Swiss-Model (https://swissmodel.expasy.org)^39–41^ (Reference PDB 3ZKB [42]), because the crystal structures for *Mle*-DNA Gyrase have not been reported. Based on the predicted structure, we performed amino acid sequence and multiple structural alignments between *Mle*- and *Mtb*-DNA GyrB (H37Rv) subunits (Suppl. Fig. 2) and identified the amino acid regions of G105-S118 and V214-R250 of the ATPase domain as the differential region in the N-terminus of the GyrB domains^42,43^ (Suppl. Fig. 2A). Based on the results of the structural comparison, it was predicted that the structure of the G105-S118 region in *Mle*-GyrB was similar to that in *Mtb*-GyrB (Suppl. Fig. 2B). On the other hand, since the structure of the V214-R250 region in *Mtb*-GyrB has not been determined, a detailed comparison cannot be made. It is possible that the structure of the V214-R250 region in *Mle*-GyrB is different from that in *Mtb*-GyrB (Suppl. Fig. 2B). However, we guess that the conformational diversity of this region cannot affect ATP binding or hydrolysis^44^ and that other structural factors might relate to the temperature sensitivity of DNA gyrase. To clear this point, the determination of the 3D structure of *Mle*-DNA gyrase is in progress.

To summarise this study, *in vitro* and *in vivo* data collectively showed that DNA gyrase activity was strongly correlated with the growth of *Mle*. Importantly, immersion in hot springs or saunas, whose temperatures are typically above 40 °C, may effectively treat or prevent leprosy because the enzyme is inactive and the bacilli grow poorly at temperatures higher than 37 °C. To the best of our knowledge, this is the first detailed study of the role of *Mle*-DNA gyrase in bacterial growth and survival. Future research should be aimed at performing crystallography-aided functional analysis of the GyrB subunit, as well as developing a standardised protocol for thermal therapy of leprosy.

## Materials and Methods

### Materials

Oligonucleotide primers were synthesized, and TOPO TA cloning (pCR4-TOPO) and nickel-nitrilotriacetic acid (Ni^+^-NTA) protein purification kits were purchased from Life Technologies (Carlsbad, CA, USA). Ampicillin and isopropyl-β-d-thiogalactopyranoside (IPTG) were purchased from Meiji Seika Kaisha, Ltd. (Tokyo, Japan) and Wako Pure Chemical Industries Ltd. (Osaka, Japan), respectively. Restriction enzymes were obtained from New England BioLabs, Inc. (Ipswich, MA, USA). The supercoiling assay kit, including supercoiled and relaxed pBR322 DNA, was purchased from John Innes Enterprises Ltd. (Norwich, United Kingdom), whereas the Complete Mini, EDTA-free Protease Inhibitor Cocktail was purchased from Roche Applied Science (Mannheim, Germany). GigaPackIII Gold Packaging Extract was procured from Stratagene (La Jolla, CA, USA).

### Bacterial strains and plasmids

*Mle* genomic DNA was obtained from the Thai-53 strain^45^ maintained at the Leprosy Research Center, National Institute of Infectious Diseases (Tokyo, Japan). *E. coli* Top-10 (Life Technologies) and DH5α were used as hosts for molecular cloning, whereas strains Rosetta-gami2 (DE3) *pLysS* and BL21 (DE3) *pLysS* were used to express *Mle* and *Mtb* GyrA and GyrB subunits from plasmids pET-20b (+) and pET-19b (Merck KGaA, Darmstadt, Germany). *Msmeg* mc^2^ 155 was used as the mycobacterial host to produce strains expressing DNA gyrase from *Mle* and *Mavi* 104 strains. The cosmid vector pYUB854 and plasmid vector phAE87, which were used to construct vectors for allelic exchange, were kindly provided by W. R. Jacobs, Jr. (Department of Microbiology and Immunology, Albert Einstein College of Medicine, New York, NY, USA).

### Molecular cloning

Wild-type *Mle*- and *Mtb*-DNA gyrase subunits were cloned as previously described^25–27^. Briefly, the genes from genomic DNA obtained from *Mle* Thai-53 and *Mtb* H37Rv strains were amplified by PCR (Applied Biosystems, Foster City, CA, USA) using previously reported primers^25–27^. PCR products were then ligated to the TA cloning plasmid and cloned in *E. coli* Top-10. *Mtb-*DNA *gyrA*, *Mle-*DNA *gyrA*, and *Mle-*DNA *gyrB* were then subcloned via *Nde*I and *Xho*I digestion into pET-20b (+). *Mtb-*DNA *gyrB* was subcloned into pET19b using the same sites. Plasmids were sequenced on an ABI Prism 3130xl Genetic Analyzer (Life Technologies) using an ABI Prism BigDye Terminator v3.1 Cycle Sequencing Kit (Life Technologies), following the manufacturer’s instructions. Sequences were compared with reference sequences using Bioedit (http://www.bioedit.com/).

### Overexpression and purification of recombinant DNA gyrase

DNA gyrase subunits were purified as previously described^25–27^. Briefly, the expression vectors carrying the recombinant *Mtb-*DNA *gyrA* and *Mle-*DNA *gyrA/B* were transformed and expressed in Rosetta-gami2 (DE3) *pLysS*, whereas *Mtb-*DNA GyrB was expressed in BL21 (DE3) *pLysS*. Transformants were grown at 37 °C for 12 h in 200-mL cultures, at which point the optical density at 600 nm was 0.8–1.0. Expression of GyrA and GyrB was then induced with 1.0 mM IPTG for 16 h at 14 °C (*Mle* subunits and *Mtb*-DNA GyrA) or for 5 h at 23 °C (*Mtb*-DNA GyrB), respectively. Subsequently, cultures were harvested by centrifugation at 22,540 × *g* at 4 °C for 10 min. The pellets were stored at −80 °C for 12 h. The pellets were then suspended in 10 mL of native binding buffer (20 mM Tris-HCl [pH 8.0], 500 mM NaCl, 10 mM imidazole, and protease inhibitor cocktail); this was followed by lysis via sonication on ice with a Sonifier 250 (Branson, Inc., Japan) operating at 30% pulsar power for 20 cycles of 40 s on/1 min off and then centrifugation at 9,400 × *g* and 4 °C for 20 min. Recombinant proteins were purified via Ni^+^-NTA agarose chromatography; dialysed against 1 L of 50 mM Tris-HCl pH 7.5, 100 mM KCl, 2 mM DTT, and 1 mM EDTA; mixed with glycerol to a final concentration of 50% w/v; and stored at −80 °C until use. Samples were assayed for enzyme activity and analysed by SDS-PAGE.

### Enzymatic analysis of recombinant DNA gyrases

Purified recombinant enzymes were assayed with a combination of GyrA and GyrB subunits for DNA supercoiling activity in 30-μL reactions containing 35 mM Tris-HCl (pH 7.5), 24 mM potassium chloride, 4 mM magnesium chloride, 2 mM dithiothreitol, 1.8 mM spermidine, 1 mM ATP, 0.1 mg/mL bovine serum albumin, 6.5% w/v glycerol, 0.3 μg relaxed pBR322 DNA, and 3 μM each of GyrA and GyrB subunits. Reactions were incubated at 30 °C and 37 °C for 1 h and then terminated by adding 30 μL of 24:1 chloroform:isoamyl alcohol and 6 μL of 5× DNA loading buffer containing 40% sucrose, 100 mM Tris-HCl (pH 7.5), 1 mM EDTA, and 0.5 μg/mL bromophenol blue. Products were resolved for 90 min at 50 mA on a 1% agarose gel in 0.5× Tris borate EDTA buffer (pH 8.3) and stained with 0.7 μg/mL ethidium bromide. Supercoiling activity was assessed by quantifying supercoiled pBR322 DNA using Molecular Analyst Software in ImageJ (http://imagej.nih.gov/ij/). To facilitate direct comparisons, enzymes were assayed in at least three replicates on the same day under identical conditions. Time courses were obtained by collecting samples between 5 and 120 min at 30 °C or 37 °C. To assess temperature sensitivity, DNA gyrases were pre-incubated at 25 °C, 30 °C, 37 °C, 40 °C, 42 °C, and 50 °C for 1 h, placed on ice for 1 h, and assayed as described for 1 h at 30 °C or 37 °C.

### Functional replacement of *Msmeg* DNA *gyrB-gyrA* genes with *Mle* or *Mavi gyrB-gyrA*

Full-length *gyrB-gyrA* genes of *Mle* and *Mavi* were amplified by PCR from *M. leprae* Thai-53 and *M. avium* 104 and cloned into pMV261. The intein-coding segment in the *Mle gyrA* gene was deleted from the plasmid using PCR; this was confirmed by sequencing. The primer list is shown in Table 1. *Msmeg* mc^2^ 155 cells were transformed with plasmids carrying the *Mle* or *Mavi gyrB-gyrA* genes. Recombinants were selected on Luria-Bertani (LB) medium containing kanamycin. Allelic exchange mutants were constructed using the temperature-sensitive mycobacteriophage method, as previously described^46^. Using the *Msmeg* mc^2^ 155 genome sequence (accession number CP000480), the upstream and downstream flanking DNA sequences were used to generate a deletion mutation in the *Msmeg gyrB-gyrA* genes. To disrupt the *gyrB-gyrA* genes, DNA segments from 990 bp upstream to 19 bp downstream of the initiation codon of *gyrB* and from 38 bp downstream to 1,026 bp downstream of the termination codon of *gyrA* were cloned directionally into the cosmid vector pYUB854. This vector contains a *res*-*hyg*-*res* cassette and a *cos* sequence for lambda phage assembly. Plasmids thus produced were digested with *Pac*I, ligated to the PH101 genomic DNA excised from plasmid phAE87 by *Pac*I digestion, and packaged using GigaPackIII Gold Packaging Extract (Stratagene). The resulting mixture was used for transduction of *E. coli* STBL2 competent cells (Life Technologies Inc.) to yield cosmid DNA. After *E. coli* was transduced and the transductants were plated on hygromycin-containing medium, plasmid DNA was prepared from pooled antibiotic-resistant transductants, and electroporated into *Msmeg* mc^2^ 155. Bacterial cells were incubated at 30 °C to produce recombinant phage. Finally, the *Msmeg* transformant carrying the *Mle* or *Mavi gyrB*-*gyrA* genes was infected with the produced temperature-sensitive phage at 33 °C to induce allelic exchange, and kanamycin- and hygromycin-resistant colonies were isolated.

**Table 1:** Primers used for this study.

Primer	Sequence^a^ (5′ to 3′)	Application
**for** ***Msmeg*** ^***b***^		
MSGBAUF	GCCTTAAGGTTCGCCGAACTGGACACCT	*gyrB-A* disruption, upstream forward
MSGBAUR	GCTCTAGATTGTTCTTCTGGGCAGCCAC	*gyrB-A* disruption, upstream reverse
MSGBADF	GCCCATGGGCTAAGGAGCTGTAGGTGAG	*gyrB-A* disruption, downstream forward
MSGBADR	GCACTAGTCCAACACCACAAACCGCATG	*gyrB-A* disruption, downstream reverse
MSGBAF	GCCATGGGGAATCGTGGTCG	detection of *gyrB-A* disruption, forward
MSGBAR	AAACCTCTTGGCCGTGTCGTGATCG	detection of *gyrB-A* disruption, reverse
**for** ***Mle*** ^***b***^		
MLGBAF	GCCAGATCTAATGACTGATATCACGCTGCC	*gyrB-A* cloning, forward
MLGBAR	GCCCTGCAGTTAACCGACACCGCCGTCGG	*gyrB-A* cloning, reverse
MLGAdel-intF	ACCGAGGCTCGGCTTACTCC	deletion of intein in *gyrA*, forward
MLGAdel-intR	ATAACGCATCGCTGCCGGTG	deletion of intein in *gyrA*, reverse
**for** ***Mavi*** ^***b***^		
MAGBAF	GCCGAATTCGTGGCTGCCCAGAAGAAGAA	*gyrB-A* cloning, forward
MAGBAR	GCCAAGCTTCTAGCCGTCCGACCCCGCGG	*gyrB-A* cloning, reverse

^a^Restriction endonuclease cleavage site are underlined.

^b^*Msmeg*, *Mle*, and *Mavi means M*. smegmatis, *M*. *leprae*, and *M*. *avium*, respectively.

### Radiorespirometry analysis

*Mle* cells were prepared as described in our previous study^29^, with slight modifications. Thermosensitivity was determined by radiorespirometry analysis according to previously published methods^28–30^, using *M. bovis* BCG Tokyo as control instead of *Mtb*. Briefly, viable *Mle* and *M. bovis* BCG cells were pre-incubated for 1 h at various temperatures between 25 °C and 50 °C, placed on ice for 1 h, and then grown in BACTEC 12B medium at 30 °C and 37 °C, respectively. Mycobacterial growth was assessed every day for 8 days by quantifying ^14^CO_2_ liberated by metabolism of ^14^C-palmitic acid substrate present in the medium. Viability is reported as the highest counts per minute (CPM) observed over the course of the experiment.

## Supplementary information

Supplementary Information_190516
