# Supplementary material for: DNA gyrase could be a crucial regulatory factor for growth and survival of Mycobacterium leprae
Source: Sci Rep. 2019 Jul 25;9:10815. doi: 10.1038/s41598-019-47364-5 (PMC6658535; doi:10.1038/s41598-019-47364-5)

Supplementary information

DNA gyrase could be a crucial regulatory factor for growth and survival of *Mycobacterium leprae*

Hyun Kim[1,2,3], Yasuo Fukutomi[4], Chie Nakajima[2,5], Youn Uck Kim[6], Shigetarou Mori[1], Keigo Shibayama[1], Noboru Nakata[3,4,*] , and Yasuhiko Suzuki[2,5,*]

Suppl. Figure Legends

Suppl. fig. 1. SDS-PAGE analysis of recombinant DNA gyrase subunits of *Mle* and *Mtb*. Each recombinant protein is shown above in the appropriate lane. The His-tagged recombinant DNA gyrases were overexpressed using an *E. coli* (Rosetta-gami2 BL21 [DE3] *pLysS* and BL21 [DE3] *pLysS*) expression system and purified by nickel resin chromatography (Ni^{+}-NTA purification system). Approximately 3 μM of each protein sample was loaded on an 8% SDS-polyacrylamide gel. Following electrophoresis, proteins were revealed by staining with Quick Coomassie Brilliant Blue. Lane M, size markers (sizes are indicated to the left in kDa); lanes 1 and 2, GyrA and GyrB subunits of *Mtb* DNA gyrases; lanes 3 and 4, GyrA and GyrB subunits of *Mle* DNA gyrases, respectively. *Mle* and *Mtb* represent *M. leprae* and *M. tuberculosis*, respectively.

Suppl. fig. 2. Predicted structure of *Mle*-DNA GyrB subunit. (A) Schematic drawing of the primary structure of *Mtb*-DNA GyrB subunit (42, 43), and the amino acid sequence alignments of *Mle*- and *Mtb*-DNA GyrB using Genetyx (https://www.genetyx.co.jp). TOPRIM and red box on the alignments indicate the topoisomerase-primase and ATPase domains, respectively. (B) The structure of *Mle*-DNA GyrB was predicted using Swiss-Model (https://swissmodel.expasy.org) (39-41). The overall structure of *Mle*- and *Mtb*-DNA GyrB subunits are depicted in cyan and green colors, respectively, in the middle. The amino acid G105-S118 region is shown in the left panel, and the V214-R250 region in the right panel. *Mle* and *Mtb* noted *Mle*-GyrB for swiss model and reference PDB 3ZKB, respectively.

Suppl. fig. 3. Thermal inactivation of *Mle*- and *Mtb*-DNA gyrases. DNA gyrases were inactivated for 1 h at various temperatures (25, 30, 37, 40, 42, and 50°C), cooled on ice for 1 h, and assayed for 1 h at 30°C (*Mle,* bottom) or 37°C (*Mtb,* top). Lanes 1, inactivation at 25°C; lanes 2, inactivation at 30°C; lanes 3, inactivation at 37°C; lanes 4, inactivation at 40°C; lanes 5, inactivation at 42°C; lanes 6, inactivation at 50°C, respectively. Lane 7-12, 2nd times assay, and lane 13-18, 3rd times assay. SC and R denotes the supercoiled and relaxed pBR322 DNA, used as a positive control. All enzyme assays were performed at least three times to confirm reproducibility.

Suppl. fig. 4. DNA supercoiling activity of various combinations of *Mle*- and *Mtb*-DNA gyrase subunits. Relaxed pBR322 DNA (0.3 μg) was incubated for 1 h at 30°C (top), 37°C (middle), and 42°C (bottom) with DNA gyrase reconstituted from 3 μM of recombinant GyrA and GyrB subunits in various combinations. Lane M, *Hind*III DNA ladder; lanes 1, *Mtb* DNA gyrase subunits; lane 2, *Mtb*-GyrA+*Mle*-GyrB subuints; lane 3, *Mtb*-GyrB+*Mle*-GyrA subuints; lane 4, *Mle* DNA gyrase subunits; lane 5-8, 2nd times of assay; lane 9-12, 3rd times of assay, respectively. SC and R denotes the supercoiled and relaxed pBR322 DNA, used as a positive control.

Suppl. Fig. 1

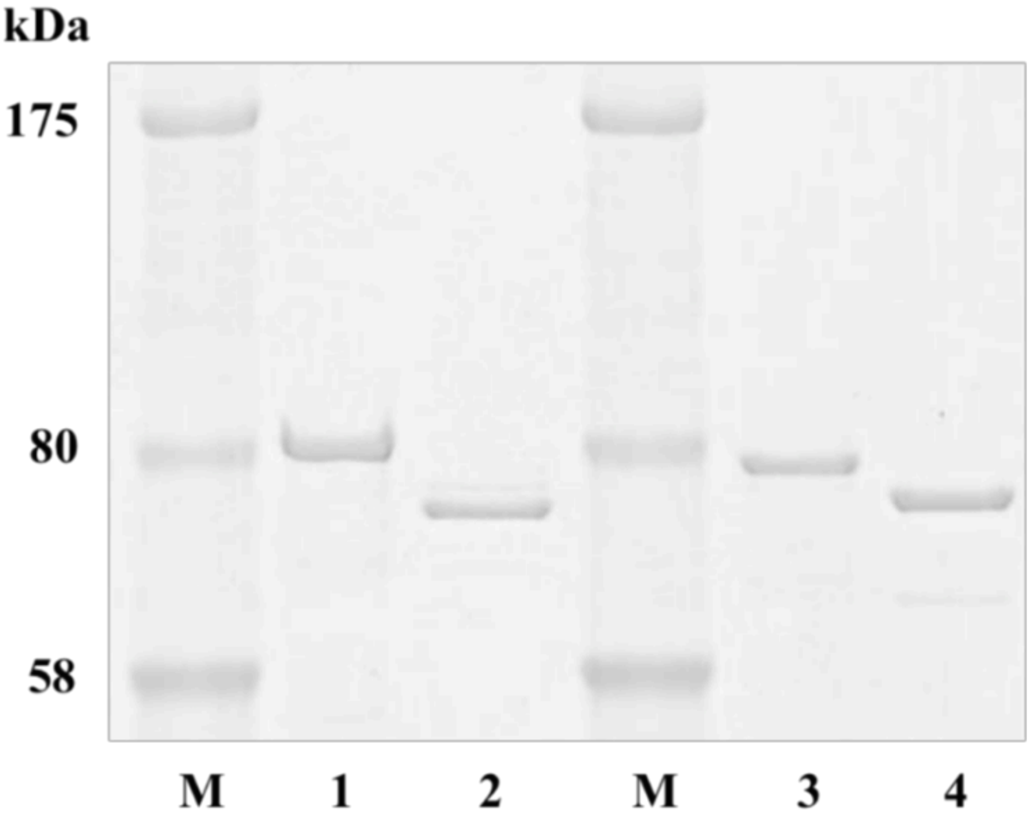

Suppl. Fig. 2

A)

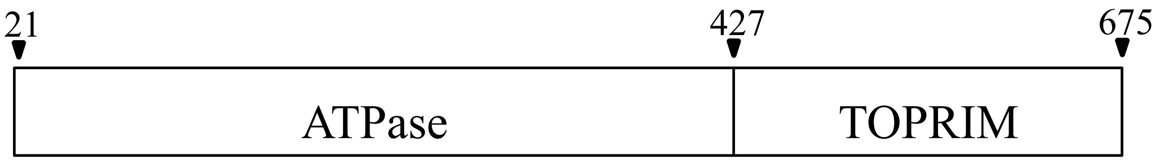


```
Mle GyrB.seq             1 ------MAAQRKAQDEYGAASITILEGLEAVRKRPGMYVGSTGERGLHHLIWEVVDNSVD    54
Mtb H37Rv GyrB.seq       1 -----VAAQKKKAQDEYGAASITILEGLEAVRKRPGMYIGSTGERGLHHLIWEVVDNAVD    55
Reference (3zkb).seq     1 GPLGSVAAQKKKAQDEYGAASITILEGLEAVRKRPGMYIGSTGERGLHHLIWEVVDNAVD    60
                                 ..*...**************************.******************.**

Mle GyrB.seq            55 EAMAGYATQVDVRLFDDGSVEVADNGRGIPVAVHATGVPTVDVVMTQLHAGGKFGGKDSG   114
Mtb H37Rv GyrB.seq      56 EAMAGYATTVNVVLLEDGGVEVADDGRGIPVATHASGIPTVDVVMTQLHAGGKFDS--DA   113
Reference (3zkb).seq    61 EAMAGYATTVNVVLLEDGGVEVADDGRGIPVATHASGIPTVDVVMTQLHAGGKFDS--DA   118
                           ********.*.*.*..**.*****.*******.**.*.****************..  ..

Mle GyrB.seq           115 YNVSGGLHGVGVSVVNALSTRVEVDIKRDGYEWSQFYDKAVPGILKQGEATEATGTTIRF   174
Mtb H37Rv GyrB.seq     114 YAISGGLHGVGVSVVNALSTRLEVEIKRDGYEWSQVYEKSEPLGLKQGAPTKKTGSTVRF   173
Reference (3zkb).seq   119 YAISGGLHGVGVSVVNALSTRLEVEIKRDGYEWSQVYEKSEPLGLKQGAPTKKTGSTVRF   178
                           *..******************.**.**********.*.*..*..****..*..**.*.**

Mle GyrB.seq           175 WADPDIFETTKYDFGTVARRIQEVAFLNKGLTINLVDERVKQDEVVDDVVSDTAEAPVAM   234
Mtb H37Rv GyrB.seq     174 WADPAVFETTEYDFETVARRLQEMAFLNKGLTINLTDERVTQDEVVDEVVSDVAEAP--K   231
Reference (3zkb).seq   179 WADPAVFETTEYDFETVARRLQEMAFLNKGLTINLTDERVTQDEVVDEVVSDVAEAP--K   236
                           ****..****.***.*****.**.***********.****.******.****.****   .

Mle GyrB.seq           235 TVEEKSTESSAPHKVRHRTFHYPGGLVDFVKHINRTKTPIQQSIIDFDGKGAGHEVEVAM   294
Mtb H37Rv GyrB.seq     232 SASERAAESTAPHKVKSRTFHYPGGLVDFVKHINRTKNAIHSSIVDFSGKGTGHEVEIAM   291
Reference (3zkb).seq   237 SASERAAESTAPHKVKSRTFHYPGGLVDFVKHINRTKNAIHSSIVDFSGKGTGHEVEIAM   296
                           ...*...**.*****..********************..*..**.**.***.*****.**

Mle GyrB.seq           295 QWNGGYSESVHTFANTINTHEGGTHEEGFRSALTSVVNKYAKDKKLLKDKDPNLTGDDIR   354
Mtb H37Rv GyrB.seq     292 QWNAGYSESVHTFANTINTHEGGTHEEGFRSALTSVVNKYAKDRKLLKDKDPNLTGDDIR   351
Reference (3zkb).seq   297 QWNAGYSESVHTFANTINTHEGGTHEEGFRSALTSVVNKYAKDRKLLKDKDPNLTGDDIR   356
                           ***.***************************************.****************

Mle GyrB.seq           355 EGLAAVISVKVSEPQFEGQTKTKLGNTEVKSFVQRVCNEQLIHWFEANPVDAKAVVNKAI   414
Mtb H37Rv GyrB.seq     352 EGLAAVISVKVSEPQFEGQTKTKLGNTEVKSFVQKVCNEQLTHWFEANPTDAKVVVNKAV   411
Reference (3zkb).seq   357 EGLAAVISVKVSEPQFEGQTKTKLGNTEVKSFVQKVCNEQLTHWFEANPTDAKVVVNKAV   416
                           **********************************.******.*******.***.*****.

Mle GyrB.seq           415 SSAQARIAARKARELVRRKSATDLGGLPGKLADCRSTDPRSSELYVVEGDSAGGSAKSGR   474
Mtb H37Rv GyrB.seq     412 SSAQARIAARKARELVRRKSATDIGGLPGKLADCRSTDPRKSELYVVEGDSAGGSAKSGR   471
Reference (3zkb).seq   417 SSAQARIAARKARELV--------------------------------------------   432
                           ****************....... ................ ..................

Mle GyrB.seq           475 DSMFQAILPLRGKIINVEKARIDRVLKNTEVQAIITALGTGIHDEFDISRLRYHKIVLMA   534
Mtb H37Rv GyrB.seq     472 DSMFQAILPLRGKIINVEKARIDRVLKNTEVQAIITALGTGIHDEFDIGKLRYHKIVLMA   531
Reference (3zkb).seq   433 ------------------------------------------------------------   432
                           ...............................................  ..........

Mle GyrB.seq           535 DADVDGQHISTLLLTLLFRFMRPLIEHGYVFLAQPPLYKLKWQRMDPEFAYSDSERDGLL   594
Mtb H37Rv GyrB.seq     532 DADVDGQHISTLLLTLLFRFMRPLIENGHVFLAQPPLYKLKWQRSDPEFAYSDRERDGLL   591
Reference (3zkb).seq   433 ------------------------------------------------------------   432
                           ......................... . ............... ........ ......

Mle GyrB.seq           595 ETGLKLGKKINKEDGIQRYKGLGEMDAKELWETTMDPSVRVLRQVTLDDAAAADELFSIL   654
Mtb H37Rv GyrB.seq     592 EAGLKAGKKINKEDGIQRYKGLGEMDAKELWETTMDPSVRVLRQVTLDDAAAADELFSIL   651
Reference (3zkb).seq   433 ------------------------------------------------------------   432
                           . ... ......................................................

Mle GyrB.seq           655 MGEDVDARRSFITRNAKDVRFLDV                                       678
Mtb H37Rv GyrB.seq     652 MGEDVDARRSFITRNAKDVRFLDV                                       675
Reference (3zkb).seq   433 ------------------------                                       432
                           ........................
```

[a] mean the ATPase domain on GyrB subunit

Suppl. Fig. 2. continue

B)

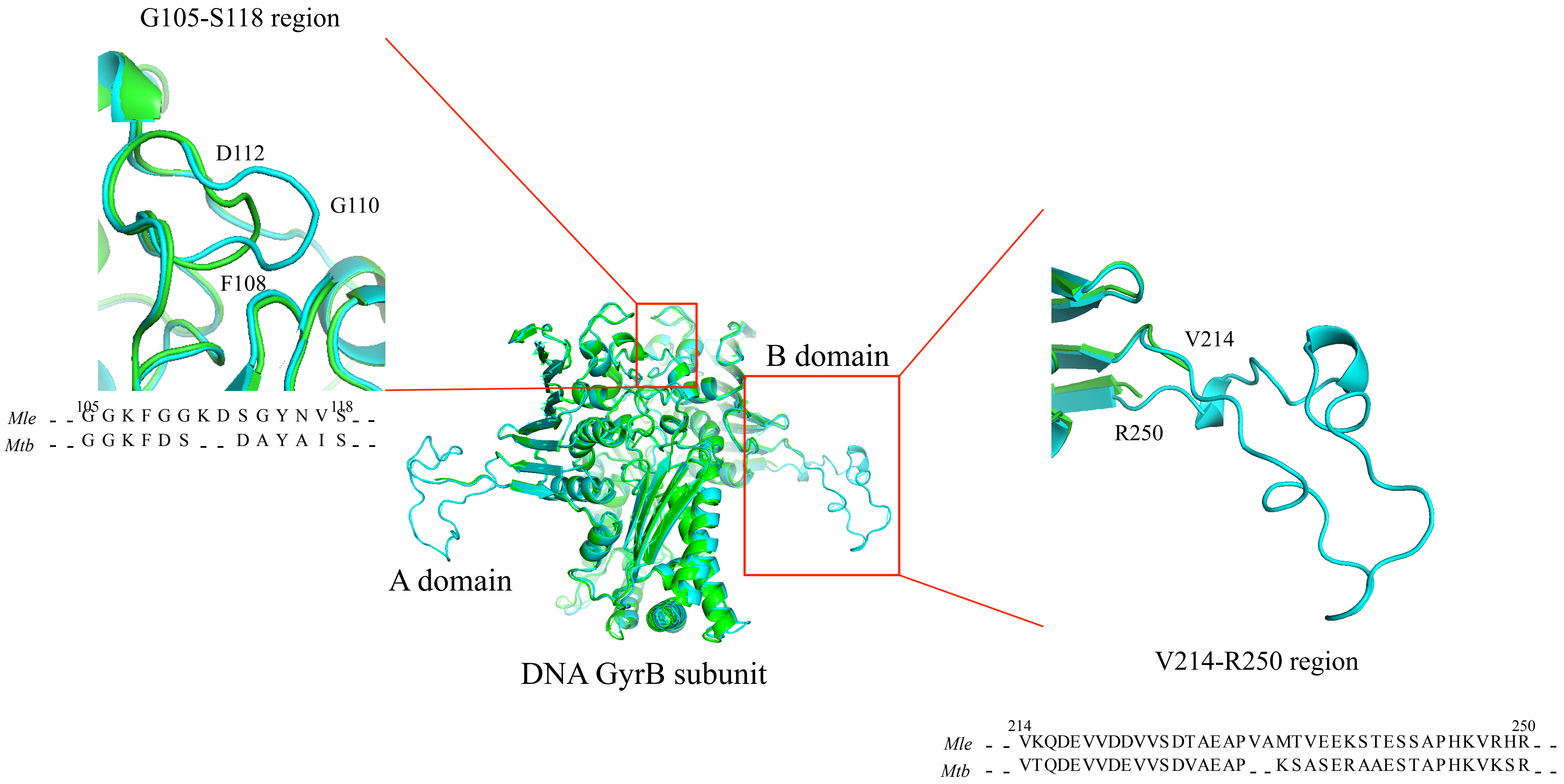

Suppl. Fig. 3

Full-length of electrophoresis for Fig. 3.

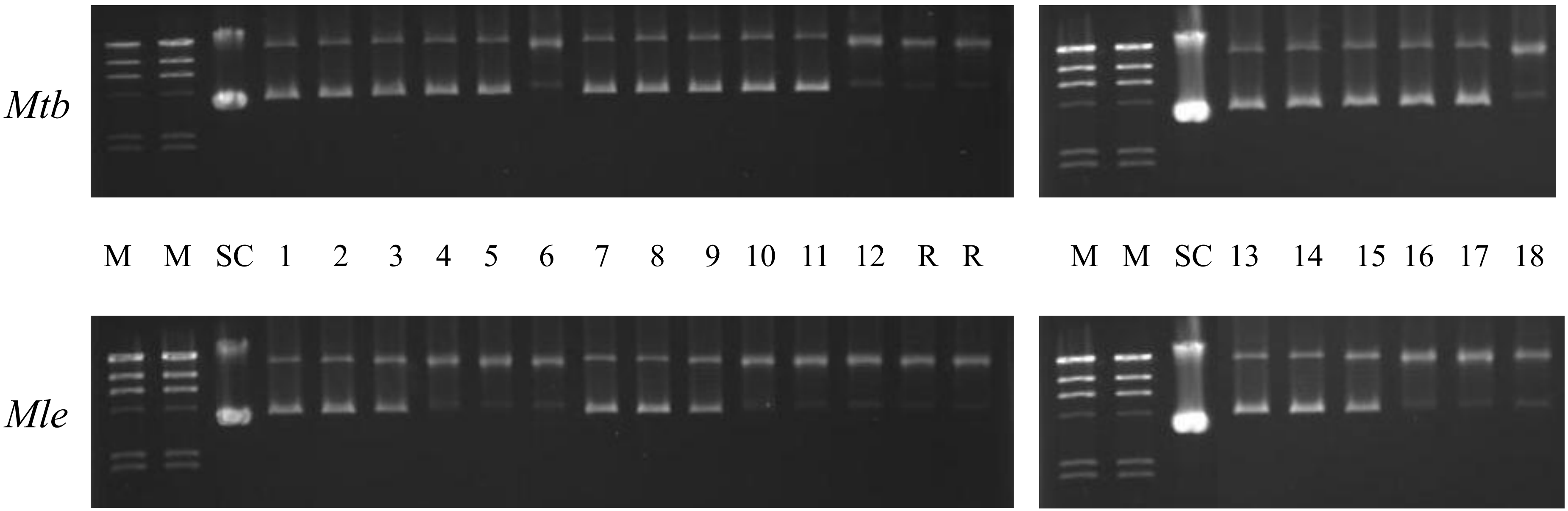

Suppl. Fig. 4.

Full-length of electrophoresis for Fig. 7.

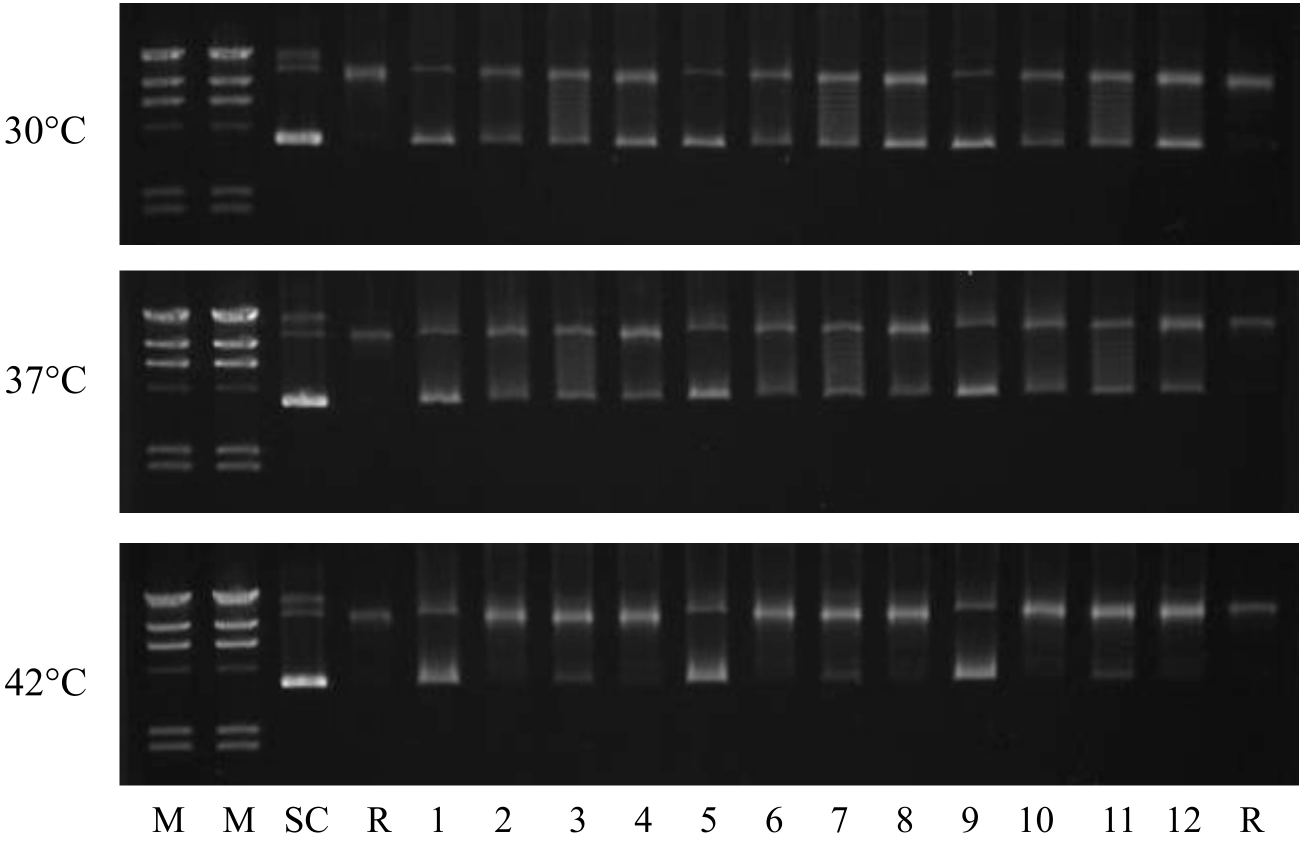

Supplement: Supplementary file 1 — Supplementary Information_190516 [file 41598_2019_47364_MOESM1_ESM.pdf]
